# Comparative analysis of the hydrogen sulphide pathway in internal thoracic artery and radial artery

**DOI:** 10.1093/icvts/ivac105

**Published:** 2022-04-21

**Authors:** Yoonjin Kang, Jun Sung Kim, Huixing Cui, Myoung-Jin Jang, Yin Hua Zhang, Ho Young Hwang

**Affiliations:** 1 Department of Thoracic and Cardiovascular Surgery, Seoul National University Hospital, Seoul, Republic of Korea; 2 Department of Thoracic and Cardiovascular Surgery, Seoul National University Bundang Hospital, Seoul National University College of Medicine, Seongnam, Republic of Korea; 3 Department of Physiology, College of Medicine, Seoul National University, Seoul, Republic of Korea; 4 Medical Research Collaborating Center, Seoul National University Hospital, Seoul National College of Medicine, Seoul, Republic of Korea

**Keywords:** Coronary artery bypass grafting, Internal thoracic artery, Radial artery, Hydrogen sulphide

## Abstract

**OBJECTIVES:**

The molecular basis supporting the superiority of the left internal thoracic artery (LITA) as a bypass conduit is limited. This study was conducted to compare the expression and localization of hydrogen sulphide synthesizing enzymes in LITA and radial artery (RA).

**METHODS:**

Nineteen patients who underwent coronary artery bypass grafting using LITA and RA were enrolled. The remnant LITA and RA were collected to measure the expression levels of 3 hydrogen sulphide-producing enzymes: cystathionine β-synthase, cystathionine γ-lyase and 3-mercaptopyruvate sulphurtransferase using quantitative real-time polymerase chain reaction. Expression levels of these enzymes in the LITA and RA were compared in each subject. The expression and localization patterns of the enzymes were also analysed by immunohistochemistry.

**RESULTS:**

The mRNA expression of the cystathionine β-synthase was greater in the LITA than in the RA (*P *=* *0.033), whereas the expression levels of the other 2 enzymes did not significantly differ between the 2 arteries. The immunohistochemistry analysis demonstrated greater expression of the cystathionine β-synthase in the LITA than in the RA (*P *=* *0.006). This protein was present in both tunica intima and tunica media of the LITA, although it was present only in the tunica media of the RA. Localization patterns of the other 2 enzymes were not different between LITA and RA.

**CONCLUSIONS:**

Expression levels of the mRNA and protein of cystathionine β-synthase were significantly greater in LITA than in the RA. These findings might be a factor that affects the superior patency rate of LITA.

**Figure:**
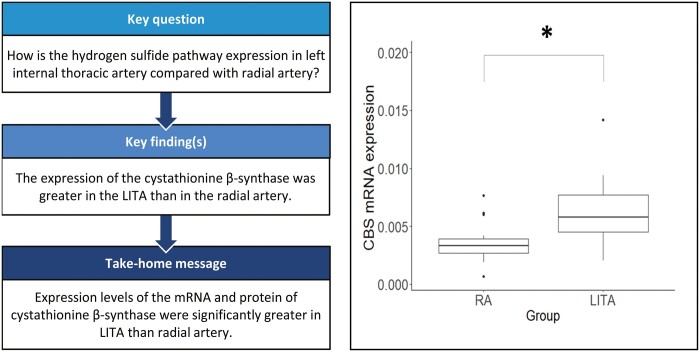

## INTRODUCTION

The internal thoracic artery (ITA) and radial artery (RA) are widely used conduits in coronary artery bypass grafting (CABG). The long-term patency and clinical outcomes after CABG using the ITAs are known to be excellent [1–3]. Previous studies suggested that the ITA is superior to other arteries in its resistance to atherosclerosis because of its abundant nitric oxide (NO) release [4–7]. However, the underlying molecular mechanism has yet to be confirmed.

Hydrogen sulphide (H_2_S) is a gasotransmitter, which is endogenously produced in 3 independent pathways, and 3 enzymes act as key enzymes in each pathway: cystathionine γ-lyase (CTH), cystathionine β-synthase (CBS) and 3-mercaptopyruvate sulphurtransferase (MPST) [8]. The vasoprotective properties of H_2_S include vasodilation, reduction of inflammation, inhibition of platelet aggregation and the scavenging of reactive oxygen species [9]. A recent study based on metabolome profiles showed greater plasma l-cysteine and H_2_S concentrations in the ITA than in the ascending aorta [10].

The present study was conducted to compare the expression and localization patterns of H_2_S-synthesizing enzymes in human ITA and RA, using quantitative real-time polymerase chain reaction (PCR) and immunohistochemistry (IHC).

## PATIENTS AND METHODS

### Ethics statement

The study protocol was reviewed by our Institutional Review Board and approved as a prospective translational research study (approval number: H-2005-040-1122). A written formal consent was obtained from the participants.

### Patient enrolment

This prospective study was conducted in patients who were scheduled for CABG using the left ITA (LITA) and the RA. A study population of 20 patients was planned. The study protocol was reviewed and approved by the Institutional Review Board at 2 participating institutions and individual consent was obtained from all study patients.

**Figure 1:**
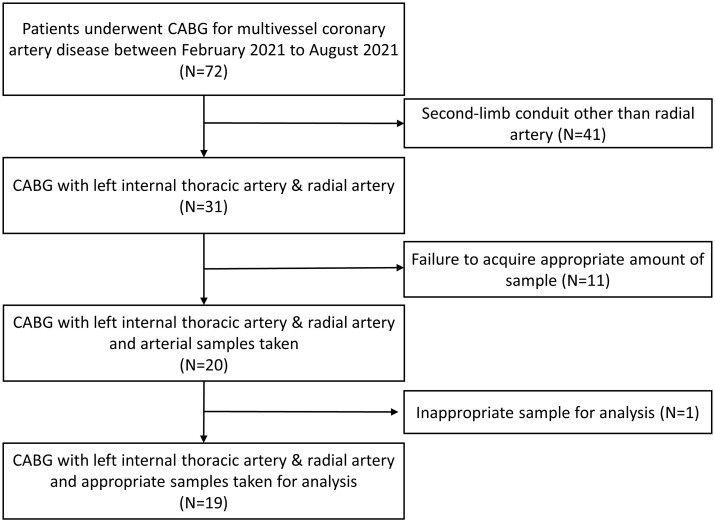
Flow diagram of patient enrolment.

Among the 99 patients who underwent CABG between February 2021 and August 2021, 96 were screened for study eligibility. Although 72 patients were eligible for study enrolment, 41 were excluded because a second-limb conduit (other than the RA) was used. Among the remaining 31 patients, 11 were excluded because the amount of sample needed for the study could not be acquired during surgery. Thus, sample tissues of the LITA and RA were obtained from 20 patients. One patient was excluded because of insufficient tissue quality with extremely low DNA concentration; 19 patients were thus included in the study (Fig. 1). The median patient age was 67 years [interquartile range (IQR) = 60, 73]; the most common comorbidities were hypertension and dyslipidaemia (Table 1). The distal ends of the LITA and RA were obtained concurrently after the arteries had been harvested and the graft lengths needed for CABG had been estimated. The mean lengths of approximately 10 mm were obtained for both the LITA and the RA. For IHC, vessel tissues of approximately 5 mm were isolated and fixed in formaldehyde buffer (4% w/v). For quantitative real-time PCR, remnant tissues were immediately stored in a portable liquid nitrogen tank.

**Table 1:** Baseline characteristics of the patients

Variables	Total (*n* = 19)
Age (years)	67 (60, 73)
Male	16 (84.2)
Risk factors	
Overweight (body mass index>25 kg/m^2^)	10 (52.6)
Smoking	14 (73.7)
Hypertension	13 (68.4)
Diabetes mellitus	10 (52.6)
Dyslipidaemia	13 (68.4)
History of stroke	0 (0)
Chronic renal failure (GFR <60 ml/min)	0 (0)
COPD	0 (0)
Peripheral vascular disease	0 (0)
Left ventricular dysfunction (LVEF <30%)	1 (5.3)
Left main disease	6 (31.6)
Three-vessel disease	19 (100)
Diagnosis	
Unstable angina	2 (10.5)
Stable angina	17 (11.8)
EuroSCORE	0.88 (0.61, 1.28)

Data are given as the median (interquartile ranges) or *n* (%).

GFR: glomerular filtration rate; LVEF: left ventricular ejection fraction; COPD: chronic obstructive pulmonary disease.

### Ribonucleic acid isolation and quantitative real-time polymerase chain reaction analysis

Total mRNA was isolated from human vessel tissues using the QIAzol lysis reagent (cat. no. 79306), in accordance with the manufacturer’s protocol. The concentration and integrity of the RNA were determined using a NanoDrop 2000/2000C spectrophotometer (Thermo Scientific, USA), with 260/280 and 230/260 nm ratios of 1.8–2.0 and 2.0–2.2 defined as acceptable. Complementary deoxyribonucleic acid (cDNA) synthesis was done with 1 μg of total RNA using a Takara prime script cDNA synthesis kit (Takara Korea Biomedical Inc, Korea). After reverse transcription had been performed, the cDNA was diluted and then amplified in a CFX96 real-time thermal cycler (Bio-Rad) using FAM (Invitrogen) as the fluorophore. The PCR conditions in the SYBR GreenGene expression assay were 3 min at 95°C, followed by 40 cycles of 10 s at 95°C and 30 s at 55°C. Assay kits for human CBS, CTH, MPST, and 18S rRNA were obtained from Assay-on-Demand (Applied Bioneer Corp., Korea). The results were calculated using the 2^–ΔΔCT^ method [11] and expressed as the fold change in the gene of interest in the treated versus control samples. All reactions were performed in triplicate, and 18S rRNA was used as an internal control.

### Immunohistochemistry analysis

Sections of formaldehyde-fixed, paraffin-embedded vessels were used for immunostaining. Tissue sections were cut, placed on slides and stained using the Discovery XT automated immunohistochemistry stainer (Ventana Medical Systems, Inc., Tucson, AZ, USA). Signals were detected using the Ventana ChromoMap kit (Ventana Medical Systems, Inc.).

The sections were deparaffinized using EZ Prep solution. A CC1 standard (Tris/borate/EDTA buffer, pH 8.4) was used for antigen retrieval. Inhibitor D (endogenous peroxidase) was blocked by incubation of the slides with 3% H_2_O_2_ for 4 min at 37°C. The slides were then incubated with primary anti-CBS (1:200), anti-CTH (1:50) and anti-MPST (1:50) antibodies for 32 min at 37°C, followed by incubation with a secondary antibody (Omimap anti-mouse HRP) for 20 min at 37°C. After an 8-min incubation in 3,3′-diaminobenzidine + H_2_O_2_ substrate at 37°C, the sections were counterstained with haematoxylin and blueing reagent at 37°C. Reaction buffer (pH 7.6 Tris buffer) was used as the washing solution.

After IHC, the slides were scanned using the Aperio ScanScope (Leica, Buffalo Grove, IL, USA). Digital images of the slides were processed using ImageScope software (Leica).

Immunoreactivity was grossly evaluated as strongly positive (>75% positive cell rate at 400× magnification), moderately positive (25–75% positive cell rate), weakly positive (<25% positive cell rate) or negative (0% positive cell rate). A semi-quantitative comparison of the IHC results was performed only for CBS, because neither CTH nor MPST showed obvious differences in immunoreactivity in the RA vs LITA.

IHC analysis was not blinded but it was performed by an automated program; the expression value was quantified using Fuji ImageJ software and normalized based on the nuclear intensity value, as previously described [12]. In brief, the software was used to perform colour deconvolution of the IHC images, after which the mean grey values were measured using deconvoluted images of 3,3′-diaminobenzidine-stained cells. The number of nuclei detectable on the slides by haematoxylin staining was determined by counting particle numbers. The mean grey values were normalized based on the number of cell nuclei.

### Statistical analysis

Statistical analyses were performed using SPSS software (version 23.0, IBM SPSS Statistics for Windows, Armonk, NY, USA) and R software (version 3.6.2, Boston, MA, USA). The data were analysed for normality using the Kolmogorov–Smirnov test. Continuous variables that were not normally distributed were expressed as medians and IQRs. Because the mRNA expression data were paired data in each patient and did not exhibit normality, they were analysed using the Wilcoxon signed rank test. *P*-value <0.05 was considered as statistically significant.

## RESULTS

### Quantitative real-time polymerase chain reaction analysis

**Figure 2:**
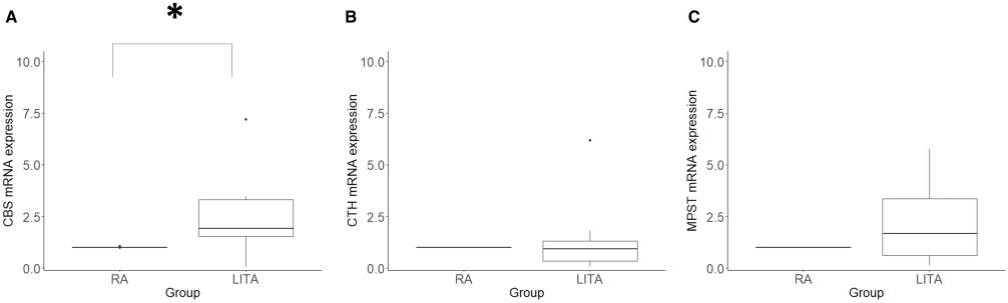
Expression of H_2_S-producing enzymes in the left internal thoracic artery and radial artery. Quantification of (**A**) cystathionine β-synthase, (**B**) cystathionine γ-lyase and (**C**) 3-mercaptopyruvate sulphurtransferase mRNA levels in the left internal thoracic artery and radial artery using quantitative real-time polymerase chain reaction. For all 3 mRNAs, expression was normalized to 18S rRNA. Data are expressed as median and interquartile. *P <* *0.05.

CBS mRNA expression was significantly greater in the LITA than in the RA (*P *=* *0.033, Fig. 2A), whereas CTH and MPST mRNA levels did not significantly differ between the 2 arteries (*P *=* *0.445 and *P *=* *0.198, respectively; Fig. 2B and C). In addition, CBS mRNA levels were 6.25-fold greater than CTH mRNA levels (*P *<* *0.001) using 2 ^ΔΔCt^ method.

### Immunohistochemistry analysis

**Figure 3:**
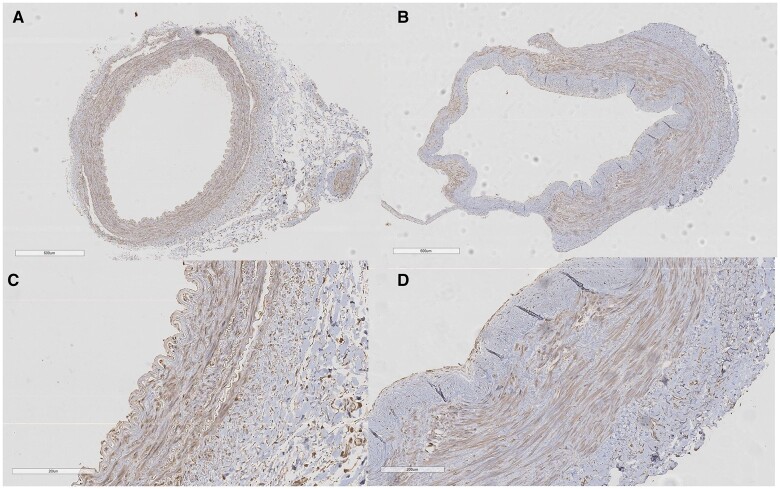
Localization of cystathionine β-synthase protein expression in the left internal thoracic artery (**A** and **C**) and radial artery (**B** and **D**). Scale bars are given in the photographs. Magnification: ×3 (**A** and **B**) and ×10 (**C** and **D**).

**Figure 4:**
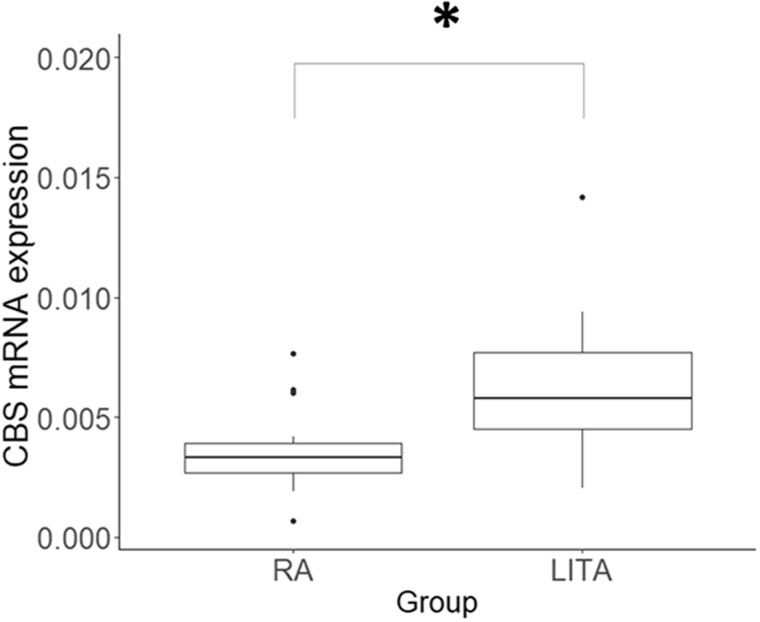
Comparison of cystathionine β-synthase protein levels in the left internal thoracic artery and radial artery via semi-quantitative immunohistochemistry analysis. Data are expressed as median and interquartile. *P <* *0.05.

**Figure 5:**
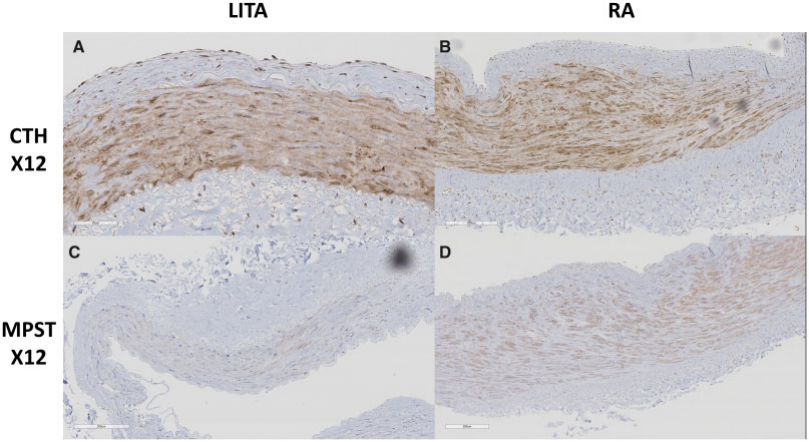
Localization of cystathionine γ-lyase (**A** and **B**) and 3-mercaptopyruvate sulphurtransferase (**C** and **D**) protein expression in the left internal thoracic artery and radial artery. Scale bars are given in the photographs. Magnification: ×12.

CBS protein expression was localized to the tunica intima and tunica media in the LITA, including endothelium layer, while it was limited to the tunica media in the RA (Fig. 3). Immunoreactivity of the CBS protein was greater in the LITA than in the RA [median (IQR) = 5.78 × 10^−3^ (4.26 × 10^−3^; 8.04 × 10^−3^) vs 3.03 × 10^−3^ (2.54 × 10^−3^; 4.04 × 10^−3^), *P *=* *0.006, Fig. 4]. On the other hand, CTH and MPST were mainly localized in the tunica media in both the LITA and RA (Fig. 5). Immunoreactivity of the CTH and MPST in both arteries was moderately positive and weakly positive, respectively (Fig. 5).

## DISCUSSION

The present study demonstrated 3 main findings. First, CBS mRNA expression was greater in the LITA than in the RA. Second, the CBS protein level was significantly greater in the LITA than in the RA. Finally, CBS protein expression was mostly localized to the endothelium in the LITA and the tunica media in the RA.

The LITA is the conduit of choice in CABG based on its excellent long-term patency and clinical outcomes [2, 13, 14]. The resistance of the ITA to atherosclerotic changes ensures its long-term patency [15–17]. A previous study showed that arteries of 350-µm thickness or 29 lamellar units are adequately perfused and nourished from the lumen [16]. Because the ITA falls within this range, it can be used as a graft without ischaemic damage to the vessel wall. Other studies have shown that the ITA endothelium is rich in heparin sulphate and endothelial NO synthase (eNOS) [4, 15]. The abundant release of NO is critical to endothelial homeostasis and the prevention of atherosclerosis [4]. However, little is known regarding the roles of molecules other than NO and heparin with respect to the mechanical properties of the ITA.

Similar to NO, H_2_S was once believed to be merely a toxic gas [18]; its important roles in vascular homeostasis, inflammation control, angiogenesis and oxidative stress are now recognized [9, 19, 20]. H_2_S-mediated protection against cardiovascular disease is facilitated by vasorelaxation, the inhibition of cardiovascular remodelling and the formation of foam cells, which together prevent the progression of atherosclerosis [9].

There is also ample evidence that H_2_S has direct benefits in ischaemic heart disease. A previous study showed lower plasma levels of H_2_S in patients with coronary artery disease than in healthy controls. In addition, plasma H_2_S levels were inversely correlated with disease severity and changes in the coronary arteries [21]. The administration of H_2_S in a mouse model of myocardial infarction was able to reduce infarct size and preserve ventricular function, further demonstrating its cardioprotective function [22].

As those 3 enzymes produce H_2_S in each independent pathway [8], the expression level and localization of the enzymes can differ according to the tissues or organs. Relative abundance of those enzymes in arterial conduits might be associated with the distinct properties and functions of the vessels. High expression of CBS in LITA may suggest potential role of CBS as a major H_2_S producing enzyme, facilitating vasodilation in response to various stimuli to endothelium. The different localization of CBS between LITA and RA can be attributed to the different histopathology of 2 arterial conduits. Histologically, LITA, an elastic artery, is different from RA in that RA is a muscular artery with large smooth muscle mass in arterial media [23]. Although there should be further evidence to support this hypothesis, the localization of CBS in the endothelium may facilitate its activation by endothelium dependent stimuli and give favourable effects to the LITA itself, coronary vessels and myocardium where the LITA supplies.

The nature of the interaction between H_2_S and NO is not well understood [20], but it may result in vasodilation in the human ITA [24]. Current studies showed that NO can be upregulated by H_2_S and vice versa [25–27]. In addition, H_2_S prevents eNOS degradation and induces NO production via eNOS phosphorylation [28, 29]. It is unknown whether NO and H_2_S interact synergistically to increase their levels and functions [30–32]. However, existing data suggest a common signalling pathway that mediates vasodilation, angiogenesis and vascular remodelling [20]. As NO–H_2_S pathways may be mutually interactive, further studies are required to understand their mutual influence and potential contribution to vascular function.

The expression of CTH and the subsequent production of H_2_S in human LITA were previously reported [24]. Webb *et al.* showed that CTH and the H_2_S, which it generates, together may play a physiological role in regulating the vasodilation. However, their study used arterial homogenates to detect both the presence of the CTH substrate l-cysteine and the generation of H_2_S. Consequently, H_2_S-producing enzymes could not be assigned to distinct layers of the arterial wall. Enzyme expression patterns in other arterial conduits were not examined.

In this study, expression patterns of the H_2_S-producing enzymes CBS, CTH and MPST were evaluated in the LITA and RA. Comparisons of the mRNA and protein levels of these enzymes in the vessels of the same patient minimized interindividual variation. To our knowledge, this is the first study to semi-quantitatively determine CBS expression in the LITA and RA using IHC.

### Limitations

There were several limitations to the present study that must be noted. First, the number of enrolled patients was small; larger studies are needed to confirm our findings. Second, the analyses were conducted *in vitro*; studies in an animal model will clarify the functions of H_2_S in the ITA. Third, subgroup analyses based on the preoperative characteristics were not performed because the number of enrolled patients was relatively small.

### CONCLUSION

In conclusion, CBS mRNA and protein levels were significantly greater in the LITA than in the RA. IHC showed that CBS was mostly localized to the tunica intima in the LITA and the tunica media in the RA. These findings may at least partly explain the superior patency of the ITA.

## Data Availability

The data underlying this article will be shared on reasonable request to the corresponding author.

## ABBREVIATIONS

CABG: Coronary artery bypass grafting
CBS: Cystathionine β-synthase
cDNA: Complementary deoxyribonucleic acid
CTH: Cystathionine γ-lyase
eNOS: Endothelial NO synthase
H2S: Hydrogen sulphide
IHC: Immunohistochemistry
IQR: Interquartile range
ITA: Internal thoracic artery
LITA: Left internal thoracic artery
MPST: 3-Mercaptopyruvate sulphurtransferase
NO: Nitric oxide
PCR: Polymerase chain reaction
RA: Radial artery
